# Efficacy of Immune Checkpoint Inhibitors in the Treatment for Head and Neck Cancer Patients: A Systematic Review and Network Meta-Analysis

**DOI:** 10.32604/or.2025.065911

**Published:** 2025-08-28

**Authors:** Jiao Li, Nurhayu Ab Rahman, Suharni Mohamad, Guang Yang, Caixia Zhao

**Affiliations:** 1 School of Dental Sciences, Universiti Sains Malaysia, Health Campus, Kubang Kerian, 16150, Malaysia; 2 Department of Pathology, Changzhi Medical College, Changzhi, 046000, China; 3 Oral Medicine and Oral Pathology Unit, School of Dental Sciences, Universiti Sains Malaysia, Health Campus, Kubang Kerian, 16150, Malaysia; 4 Department of General Surgery, Ruijin Hospital, Shanghai Jiao Tong University School of Medicine, Shanghai, 200000, China

**Keywords:** Head and neck squamous cell carcinoma, immune checkpoint inhibitor, Programmed Death-1, Programmed Death-Ligand 1, network meta-analysis

## Abstract

**Objectives:**

Checkpoint inhibitors have significantly improved outcomes in a number of malignancies. To determine the most effective course of treatment for head and neck squamous cell carcinoma (HNSCC), this systematic review evaluated the efficacy of several therapeutic approaches based on immune checkpoint inhibitors (ICIs).

**Methods:**

A comprehensive evaluation of the literature was conducted, looking at randomized controlled trials (RCTs) that were published in Embase, PubMed, and the Cochrane Central Register of Controlled Trials since database establishment. The risk of bias of the enrolled studies was analyzed using The Review Manager (RevMan) 5.4. Using network meta-analyses (NMA), the relative treatment effects on overall survival (OS) and progression-free survival (PFS) from qualifying randomized controlled trials were synthesized and evaluated.

**Results:**

Regarding OS, compared with nivolumab plus chemotherapy, chemotherapy (Hazard ratio (HR) = 2.1, 95% Confidence interval (CI): 1.2, 3.4) showed a treatment disadvantage. Meanwhile, nivolumab plus chemotherapy may represent the most efficient (57.89%) and has a lower cost among all the treatments enrolled in this study for advanced HNSCC. Regarding PFS, compared with nivolumab plus ipilimumab, nivolumab plus chemotherapy (HR = 0.4, 95% CI: 0.2, 0.8) showed treatment superiority. Additionally, nivolumab plus chemotherapy (77.18%) has the longest PFS among all interventions.

**Conclusion:**

Taking into account OS and PFS, the combination of nivolumab plus chemotherapy may appear to be the most effective option and is associated with a comparatively lower cost among all treatments included in this network meta-analysis, thereby recommending its use as a first-line therapy for HNSCC.

**Registration:**

INPLASY (2024070073).

## 1 Introduction

Head and neck cancer (HNC) currently ranks sixth in both incidence and mortality rates in the world in 2022 [1], which include cancer of the mouth, lip, nose, oropharynx, throat, and nasopharynx [2]. Furthermore, 90% of instances of HNC are head and neck squamous cell carcinoma (HNSCC) [3]. Risk variables for HNSCC consist of lifestyles, the history of infection with human papillomavirus (HPV) or Epstein-Barr virus (EBV), and tobacco and alcohol use [4,5]. The treatment strategy for HNSCC patients relies on the disease stage, the presence of lymph node metastases, distant metastases, anatomical location, and surgical accessibility. Treatment for HNSCC is complex and subtype-dependent, which typically involves surgical resection of the tumor followed by ionizing radiation (IR) therapy or chemoradiotherapy [6,7]. Conversely, approximately 50%–60% of patients with HNSCC experience short survival along with locally progressed or metastatic disease [8,9]. This could account for the total global survival rate of only 50% in HNSCC [10].

Immune checkpoint inhibitors (ICIs) have demonstrated promising effectiveness in the treatment of patients with HNSCC, especially in those who have progressed after platinum-based therapies. For advanced HNSCC patients, novel immunotherapy agents—antibodies that target the programmed death-1 (PD-1)/programmed death-ligand 1 (PD-L1) system—offer improved effectiveness and relatively less toxicity as compared to standard therapies [11–13]. Initial ICIs licensed to treat HNSCC resistant to platinum, recurrent/metastatic (R/M) are the monoclonal antibodies the anti-PD-1 pembrolizumab and nivolumab [11,13]. Pembrolizumab was cleared for use in the initial line of treatment for patients whose tumors have a PD-L1 combined positive score (CPS) of ≥1% as per the outcomes of KEYNOTE-048 research, either together with cisplatin/5-fluorouracil alone or with chemotherapy [14]. These immunotherapeutic medicines increase immune system responsiveness by blocking inhibitory signals through the PD-1/PD-L1 pathway [15,16]. But in fact, access to these regimens is limited to 5% globally [17], and the overall response is still limited.

Although ICIs are now used to treat recurrent/metastatic (R/M) HNSCC patients who have already undergone systematic chemotherapy, there is currently little pertinent data available. To the best of our knowledge, there has been only a single network meta-analysis (NMA) that compares immune checkpoint inhibitors to other systemic therapies for advanced head and neck cancer [18]. The other NMA, which included five randomized controlled trials (RCTs) (Keynote 040 and 048, Eagle, Checkmate 141, and Condor), compared anti-PD-1 to anti-PD-L1 therapies in HNSCC [19]. However, as more clinical research is ongoing, new ICIs have been involved in the treatment of HNSCC, such as ipilimumab. Added assessments are required because there aren’t enough head-to-head comparative trials to figure out whether ICI-based treatment strategies—monotherapy and combination therapy—offer the highest level of efficacy. The current systematic review examined all of the treatments, such as monotherapies, ICI combination therapies, or in conjunction with other anticancer treatments, including chemotherapy or radiation therapy in HNSCC. By synthesizing the survival advantages, including Overall Survival (OS) and Progression Free Survival (PFS), it may provide guidance for the selection or the study design of RCTs for HNSCC treatments in the future.

## 2 Methods

### 2.1 Data Sources & Search Methods

**Figure 1:**
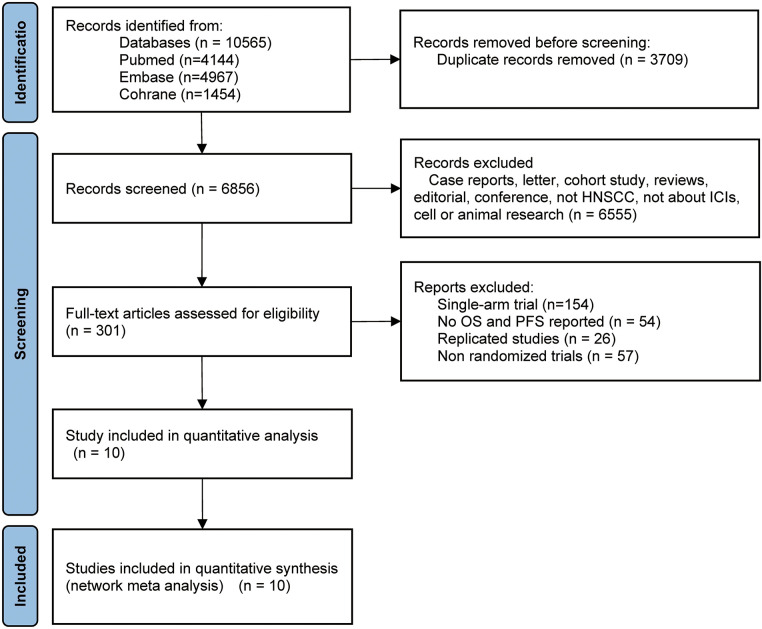
Flow diagram of study selection. HNSCC, head and neck squamous cell carcinoma; ICIs, immune checkpoint inhibitors; OS, overall survival; PFS, progression-free survival

The comprehensive declaration for network meta-analyses outlined in the PRISMA guidelines was observed during the implementation of the network meta-analysis [20]. A thorough search was conducted in the databases of Embase, PubMed, and the Cochrane Library to identify publications on immune checkpoint inhibitor therapy for HNSCC up until April 2024. A completed PRISMA 2020 checklist was used (Supplementary Material S1) to outline this study’s methodology (Fig. 1). When accessible, a search method made up of free words, Boolean search strings, and medical subject headings (MeSH terms) was used (Supplementary Tables S1–3).

### 2.2 Inclusion & Exclusion Criterion

Any research conforming to the following criterion was included: (1) the participants were adult patients (≥18 years old) diagnosed with HNC based on pathological results; (2) the intervention was ICIs of any format; (3) the study type was an RCT, blind or not; (4) the outcome contains OS and/or PFS.

The exclusion criteria consisted of the following: (1) studies that were not published in either English; (2) studies that were reviews, study protocols, conference abstracts, or duplicate publications; (3) research for which the complete text was lacking; plus (4) research that provided unextractable or insufficient data for further analysis.

### 2.3 Definition of Outcomes

OS, which refers to the time from the start of randomization (or the beginning of treatment in a single-arm trial) to death due to any cause. PFS which refers to the period of time from the start of randomization to the occurrence of disease progression or death due to any reason.

### 2.4 Search Outcomes

The bibliography was managed, and duplicates were removed using EndNote 20.4 (Clarivate Analytics, Philadelphia, PA, USA). The name of the first author, the year of publication, the patient number per arm, the course of therapy, the OS, and the PFS were all separately retrieved by two investigators, Jiao Li and Caixia Zhao. Overall Survival and Progression-Free Survival, respectively, were designated as the primary and secondary endpoints. Getting survival results together and doing a thorough analysis of them was the main goal. The 95% confidence interval (95% CI) and hazard ratios (HRs) for the time-to-event outcomes were also obtained. If the 95% CI lies entirely on one side of 1 (e.g., entirely less than 1 or entirely more than 1), then it can be confirmed that there is a significant risk difference between the experimental group and the control group. When HR > 1, the risk of the experimental group was higher than that of the control group. When HR < 1, the risk of the experimental group was lower than that of the control group. And when HR = 1, it indicates the same risk between the two groups. These 95% CI and HR results were displayed by forest plots. An agreement between the investigators settled all the disagreements on the extraction of data.

### 2.5 Evaluation of Potential Bias

Two investigators (Jiao Li and Caixia Zhao) assessed the risk of bias in the chosen research using “The Review Manager (RevMan) [Computer program] Version 5.4. The Cochrane Collaboration, 2020, for assessing the risk of bias in randomized trials” [21]. Each study’s risk of bias was evaluated separately by two investigators. All investigators came to a consensus to settle disagreements.

### 2.6 Synthesis and Statistical Analysis

The R 4.4.2 “gemtc” package was utilized for the data analysis. In all studies considered, the effect size was assessed through the use of the HRs and 95% CI. The pre-iteration and iteration count for network meta-analysis were set at 20,000 and 100,000, respectively. The Rank Probability function was used to create therapeutic impact probability ranking diagrams.

## 3 Results

### 3.1 Study Characteristics

After the databases were searched, 2388 publications in all were found (Fig. 1). A full-text review of the publications was conducted, and 10 of them were included in the study after deleting unnecessary repetition and incorporating fresh articles from cited sources. In summary, 4735 research participants were designated to receive chemotherapy, durvalumab, durvalumab + tremelimumab, nivolumab, nivolumab + chemotherapy, nivolumab + ipilimumab, nivolumab + radiotherapy, pembrolizumab, pembrolizumab + chemotherapy, tremelimumab. Table 1 provides a synopsis of the features of this research, while Fig. 1 shows the flow diagram of the study selection process.

**Table 1:** Baseline characteristics of studies included in the network meta-analysis

Authors/Year	Trial/Region	Sample size	Treatment	Number	Patients
Psyrri et al./2023 [22]	KESTREL/Globally	806	durvalumab	204	R/M HNSCC
			durvalumab + tremelimumab	413	
			chemotherapy	206	
Haddad et al./2023 [23]	CheckMate 651/Globally	947	nivolumab + ipilimumab	472	R/M HNSCC
			chemotherapy	475	
Patil et al./2023 [17]	NA/NA	151	chemotherapy	75	advanced HNSCC
			nivolumab + chemotherapy	76	
Harrington et al./2023 [24]	KEYNOTE-048/Globally	882	pembrolizumab	301	R/M HNSCC
			pembrolizumab + chemotherapy	281	
			chemotherapy	300	
Schoenfeld et al./2020 [25]	NA/United States	29	nivolumab	14	OSCC
			nivolumab + ipilimumab	15	
McBride et al./2021 [26]	NA/United States	62	nivolumab	30	R/M HNSCC
			nivolumab + radiotherapy	32	
Ferris et al./2020 [27]	EAGLE/Globally	736	durvalumab	240	R/M HNSCC
			durvalumab + tremelimumab	247	
			chemotherapy	249	
Cohen et al./2019 [13]	KEYNOTE-040/Globally	495	pembrolizumab	247	R/M HNSCC
			chemotherapy	248	
Siu et al./2019 [28]	CONDOR/Globally	267	durvalumab + tremelimumab	129	R/M HNSCC
			durvalumab	65	
			tremelimumab	63	
Ferris et al./2018 [29]	CheckMate 141/Globally	361	nivolumab	240	R/M HNSCC
			chemotherapy	121	

Note: NA, not applicable; R/M, recurrent/metastatic; HNSCC, head and neck squamous cell carcinoma; OSCC, oral squamous cell carcinoma.

### 3.2 Potential of Bias and Literature Results

**Figure 2:**
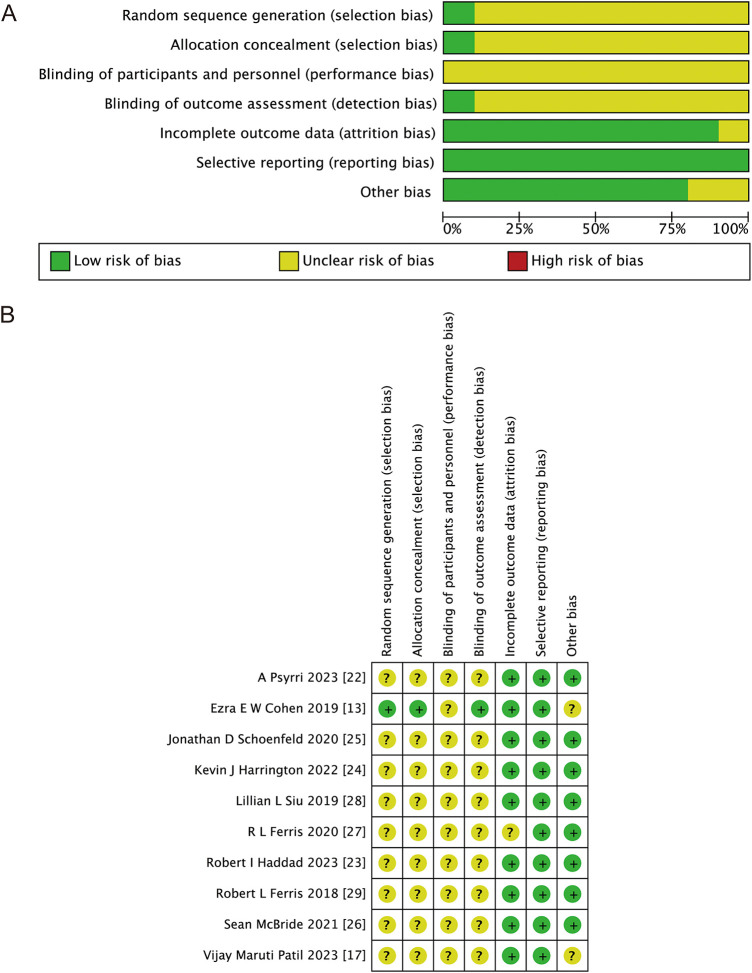
Bias risk assessment criteria for randomized controlled trials based on the Cochrane Collaborative Network. (**A**) Risk of bias graph; (**B**) risk of bias summary. Green represents low risk, yellow represents unclear risk, and red represents high risk [13,17,22–29]

Fig. 2 illustrates the risk of bias in RCTs. One study employed randomized grouping techniques: an interactive voice-response and integrated web-response system; the remaining studies did not state how the assignments were made at random after checking the main text and the supplementary documents. One research study used a third-party blind assessment of the results; the remaining studies lack any particular explanation. Most of the studies reported complete data, and all of the studies didn’t performed selective reporting. Nine and ten studies, respectively, reported PFS and OS among all the included studies. In the risk of bias assessment process, the higher the proportion of green, the higher the quality of the included articles; the higher the proportion of red, the lower the quality of the included articles; and yellow represents that the content is not obviously mentioned in the article. Therefore, Fig. 2 shows that the quality of the articles included in this study is good.

**Figure 3:**
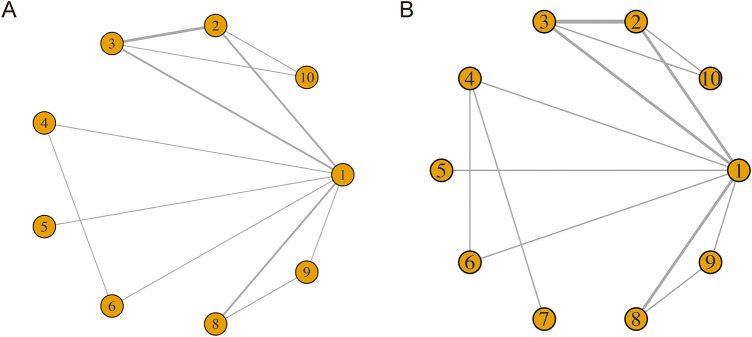
Network for PFS (**A**) and OS (**B**) on each therapeutic intervention of HNSCC. 1. chemotherapy; 2. durvalumab; 3. durvalumab + tremelimumab; 4. nivolumab; 5. nivolumab + chemotherapy; 6. nivolumab + ipilimumab; 7. nivolumab + radiotherapy; 8. pembrolizumab; 9. pembrolizumab + chemotherapy; 10. tremelimumab

Fig. 3 displays the unique network map of analogies for every result in this network meta-analysis. From Fig. 3A,B, it can be seen that there are lines between treatments 1–6, 8–10 and 1–10, respectively. The lines between 1 and 2, 2 and 3, 1 and 3, 1 and 8 in both Fig. 3A,B are much thicker than the others, indicating that there are more direct comparisons between interventions 1 and 2, 2 and 3, 1 and 3, 1 and 8 than others.

### 3.3 Progression-Free Survival (PFS) Results

**Figure 4:**
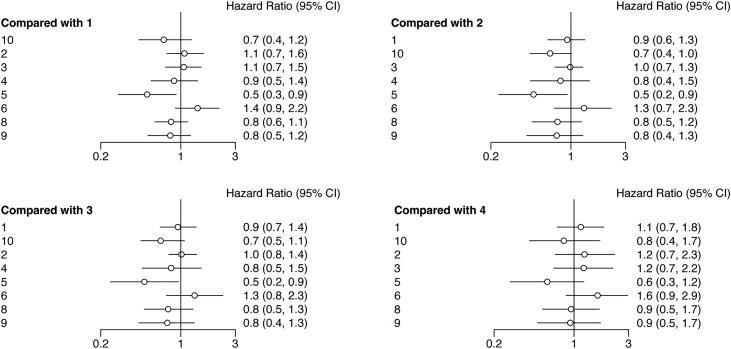

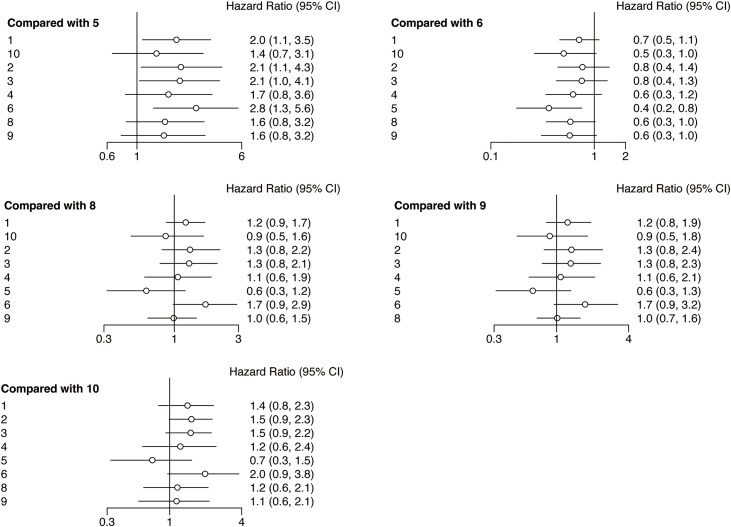
Forest plots of relative effects for PFS. Note: 1. chemotherapy; 2. durvalumab; 3. durvalumab + tremelimumab; 4. nivolumab; 5. nivolumab + chemotherapy; 6. nivolumab + ipilimumab; 7. nivolumab + radiotherapy; 8. pembrolizumab; 9. pembrolizumab + chemotherapy; 10. tremelimumab. The 95% CI did not include 1, indicating that the difference was statistically significant

Following the network analysis, a forest plot was created to illustrate the HR and 95% CIs for the comparisons among the interventions. From the forest plot, it can be estimated whether the outcome indicator is an adverse or protective factor. As presented in Fig. 4, the network meta-analysis of different interventions for improving the PFS for HNSCC patients suggested that relative to nivolumab + chemotherapy, chemotherapy (HR = 2.0, 95% CI: 1.1, 3.5), durvalumab (HR = 2.1, 95% CI: 1.1, 4.3), and nivolumab + ipilimumab (HR = 2.8, 95% CI: 1.3, 5.6) showed treatment disadvantage. And also, compared to durvalumab + tremelimumab, nivolumab + chemotherapy (HR = 0.5, 95% CI: 0.2, 0.9) showed treatment superiority.

**Figure 5:**
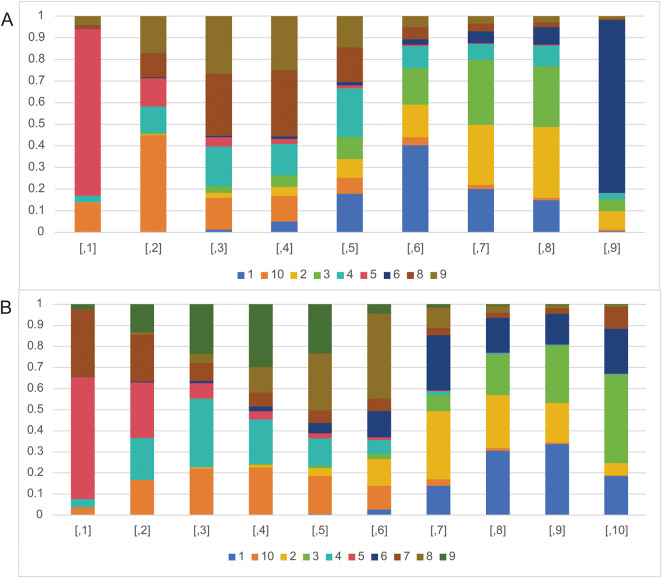
The rank probabilities of the studied interventions. (**A**) Rank probabilities of the studied interventions for PFS. (**B**) Rank probabilities of the studied interventions for OS. Note: 1. chemotherapy; 2. durvalumab; 3. durvalumab + tremelimumab; 4. nivolumab; 5. nivolumab + chemotherapy; 6. nivolumab + ipilimumab; 7. nivolumab + radiotherapy; 8. pembrolizumab; 9. pembrolizumab + chemotherapy; 10. tremelimumab

The rank probability analysis can show the probability that an intervention is ranked in a particular order among all the interventions, so as to judge the merits of an intervention in the included literature. As can be seen in Fig. 5A, nivolumab plus chemotherapy (77.18%) has the longest PFS in all interventions. Nivolumab + chemotherapy, along with tremelimumab (44.78%), pembrolizumab (28.59%), pembrolizumab + chemotherapy (25.04%), nivolumab (22.43%) have longer PFS compared with chemotherapy (40.23%). However, durvalumab + tremelimumab (29.98%), durvalumab (32.81%), and nivolumab + ipilimumab (79.84%) have shorter PFS compared with chemotherapy.

### 3.4 Overall Survival (OS) Results

**Figure 6:**
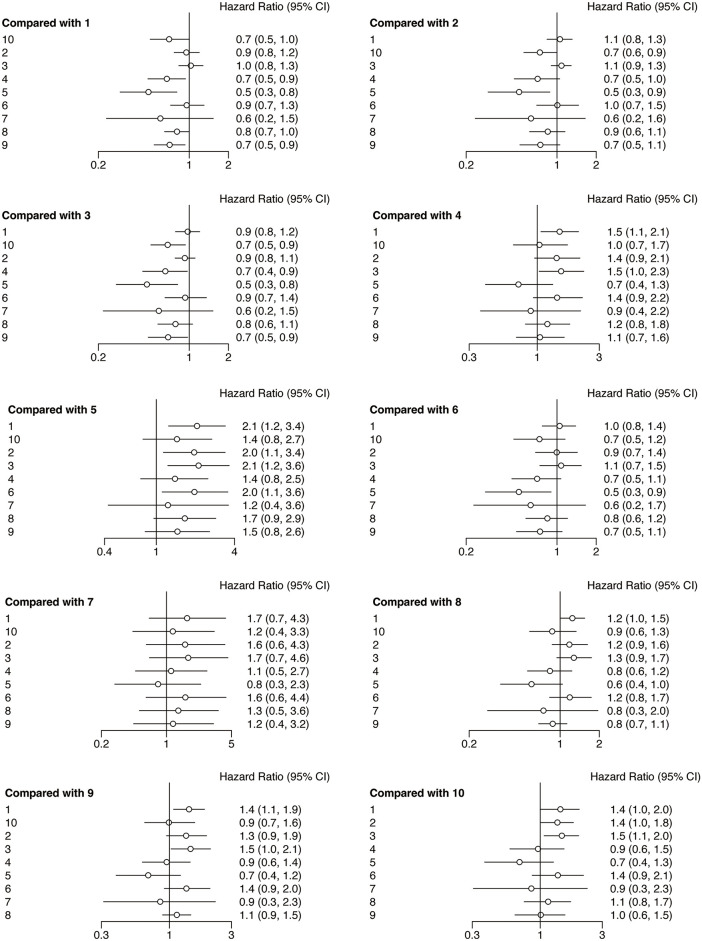
Forest plots of relative effects for OS. Note: 1. chemotherapy; 2. durvalumab; 3. durvalumab + tremelimumab; 4. nivolumab; 5. nivolumab + chemotherapy; 6. nivolumab + ipilimumab; 7. nivolumab + radiotherapy; 8. pembrolizumab; 9. pembrolizumab + chemotherapy; 10. tremelimumab. The 95% CI did not include 1, indicating that the difference was statistically significant

The forest plots of OS were illustrated in Fig. 6. The network meta-analysis examining various interventions aimed at enhancing OS for HNSCC patients indicated that, compared with the chemotherapy, nivolumab (HR = 0.7, 95% CI: 0.5, 0.9), nivolumab + chemotherapy (HR = 0.5, 95% CI: 0.3, 0.8), and pembrolizumab + chemotherapy (HR = 0.7, 95% CI: 0.5, 0.9) groups showed treatment advantage. Besides, compared with durvalumab, nivolumab + chemotherapy (HR = 0.5, 95% CI: 0.3, 0.9), tremelimumab (HR = 0.7, 95% CI: 0.6, 0.9) showed a treatment advantage. Whats more, compared with durvalumab + tremelimumab, nivolumab (HR = 0.7, 95% CI: 0.4, 0.9) nivolumab + chemotherapy (HR = 0.5, 95% CI: 0.3, 0.8), pembrolizumab + chemotherapy (HR = 0.7, 95% CI: 0.5, 0.9) and tremelimumab (HR = 0.7, 95% CI: 0.5, 0.9) showed treatment superiority. Apart from that, compared with the nivolumab + ipilimumab, nivolumab + chemotherapy (HR = 0.5, 95% CI: 0.3, 0.9) showed a treatment advantage.

The analysis of rank probability for OS indicated a predilection for nivolumab + chemotherapy (57.89%) over nivolumab + radiotherapy (22.24%). Close behind nivolumab + radiotherapy, nivolumab (32.43%), pembrolizumab + chemotherapy (29.79%), pembrolizumab (27.05%), durvalumab (12.65%), nivolumab + ipilimumab (12.51%) have better OS compared with chemotherapy (30.58%). While durvalumab + tremelimumab (27.09%) and tremelimumab (0.51%) have worse OS compared with chemotherapy (Fig. 5B).

## 4 Discussion

In the last ten years, checkpoint inhibitors have significantly improved outcomes for a number of malignancies in the palliative, adjuvant, and neoadjuvant settings [30]. The development of novel immunotherapeutic agents has led to a rapid evolution of the therapeutic arsenal for HNSCC. These medicines have been demonstrated to enhance the results of medical interventions, including PFS and OS in cancer patients, both when used together with chemotherapy and alone. The current systematic review and NMA conducted a comprehensive evaluation of the comparative effectiveness of different treatments utilising ICIs for HNSCC patients. It is true that for some HNSCC patients, ICIs improve the clinical outcome. Still, there are a lot of interventions that don’t significantly differ from conventional therapies. Therefore, this study aimed to examine the use of ICI-based therapy in HNSCC patients in an indirect manner within the context of NMA, comparing both combination and monotherapy.

### 4.1 The ICIs with Chemotherapy or Radiotherapy in HNSCC Treatment

The findings of the current NMA indicate that ICIs with chemotherapy can effectively treat HNSCC, with positive results seen in both PFS and OS. The earlier two NMAs did not compare ICI plus chemotherapy to alternative treatments [18,19]. Nivolumab plus chemotherapy provides the best PFS and OS of all the evaluated therapies; it outperforms nivolumab monotherapy. Integrating low-dose nivolumab with chemotherapy in HNSCC patients resulted in an absolute improvement in clinical outcome and reduced therapy costs to 5%–9% of full-dose immunotherapy regimens [17]. Additionally, patients who had previously received treatment in the nivolumab plus chemotherapy group demonstrated a noticeably superior clinical effect. This may be the consequence of chemotherapy-induced elevation of PD-L1 expression on tumor-infiltrating ICIs in HNSCC [31]. In comparison to earlier trials, this study’s patient sample contains fewer people with metastatic diseases, as well as those who are nonrefractory to platinum. This could result in some experimental bias, and more specific participants are needed to find out whether nivolumab plus chemotherapy has any therapeutic benefits. Aside from the aforementioned, adding low-dose nivolumab to chemotherapy is unquestionably a wise decision when taking treatment costs into account.

Furthermore, nivolumab with radiotherapy outperformed those receiving monotherapy, despite the fact that not all HNSCC patients saw an abscopal effect or an improvement in response when nivolumab and radiotherapy were combined. Nevertheless, patients with PD-L1 positive status had considerably greater OS when PD-L1 status was taken into account [26]. Radiotherapy is able to induce systemic immunity resulting from the irradiation of tumor cells immunogenic death, which can lead to subsequent anticancer responses in non-irradiated areas, like metastases. For these patients, this technique would be promising [32]. As a result, nivolumab combined with chemotherapy is the preferred choice for individuals with advanced HNSCC, particularly those who have previously received treatment. Also available to patients who test positive for PD-L1 is nivolumab with radiation therapy.

According to the findings of this study, pembrolizumab with chemotherapy outperforms treatment alone. According to the most recent NMA data, pembrolizumab monotherapy is virtually as effective as pembrolizumab combined with chemotherapy. This implies that the requirement for pembrolizumab with chemotherapy should be taken into account, and further study is required to investigate it.

### 4.2 The ICI Monotherapy in HNSCC Treatment

Regarding ICI monotherapy, pembrolizumab and nivolumab are suggested as second-line treatment of R/M HNSCC following improvement on or after platinum-containing treatment by the Food and Drug Administration (FDA) [33,34]. ICIs may make a tumor more sensitive to later treatments. Patients undergoing first-line pembrolizumab-based treatment seemed to gain from treatment using later taxanes, based on subgroup analysis categorized by the type of therapy used in the next treatment phase [24]. The current NMA findings for pembrolizumab and nivolumab are in line with earlier studies. If cost is not an issue, pembrolizumab or nivolumab monotherapy may be a considerable way to do ICI monotherapy treatment for advanced HNSCC patients, particularly PD-L1 positive patients.

However, tremelimumab was determined to be the least effective treatment regimen for overall survival (OS) based on the current results, which is in line with earlier NMA [18]. Tremelimumab, being an IgG2 monoclonal antibody, does not cause the destruction of regulatory T cells through antibody-dependent cell-mediated cytotoxicity, unlike ipilimumab [35], which could account for its lack of effectiveness. A Phase II Open-Label RCT found that although ipilimumab was administered only to HNSCC patients for a single cycle at a lower dosage, nivolumab plus ipilimumab generated many responses, including several pathologic near-complete or complete responses [25]. Further examination is necessary to establish the optimal dosage of ipilimumab and to assess the effects of tremelimumab on patients with HNSCC. Furthermore, promising PFS close for tremelimumab follows nivolumab plus chemotherapy, according to the current NMA. The reason for this could be that this NMA only contained one trial on tremelimumab monotherapy.

### 4.3 The ICIs Combination Therapy in HNSCC Treatment

The role of pembrolizumab and nivolumab in HNSCC treatment is self-evident [24,29]. Regretfully, pembrolizumab and nivolumab’s high cost prevents them from being readily available in low- and middle-income nations, which causes severe financial hardship and misery for cancer patients who are already impoverished [36,37]. But still, pembrolizumab and nivolumab remain the preferred treatments for patients with HNSCC, particularly those who test positive for PD-L1. However, because CTL penetration into the tumor is limited, many times, ICIs are useless against cold tumors, causing noticeably decreased response rates [38]. Alternative approaches such as indoximod, photodynamic treatment, oncolytic viruses, and epigenetic modification inhibitors are being studied to find new ways to improve the way that pembrolizumab and nivolumab target cold tumors [38,39].

Except for inhibitory mediators that solely target PD-1/PD-L1, the roles of the PD-L1/PD-1 and CTLA-4 pathways are essentially non-redundant, indicating that inhibiting one or the other may have synergistic or additive effects [40]. In light of this, the combined use of ICI therapy can somewhat enhance the clinical result of HNSCC. Firstly, nivolumab with ipilimumab had lower OS but substantially better PFS in OSCC that was involved in the present study [25]. In the phase III HIMALAYA trial (NCT03298451), durvalumab plus tremelimumab outperformed sorafenib in treating unresectable advanced HCC [41]. Similarly, in patients suffering from EGFR/ALK wild-type metastatic non-small-cell lung carcinoma, durvalumab and tremelimumab together with chemotherapy showed better OS and PFS benefit relative to chemotherapy in the phase III POSEIDON trial (NCT03164616) [42]. Furthermore, dual blocking of the CTLA-4 and PD-1 immune checkpoints in the neoadjuvant setting with nivolumab and ipilimumab was linked to increased pathologic response and objective response in melanoma [43,44].

The KESTREL, EAGLE, and CONDOR studies were designed for HNSCC to examine the combination of anti-PD-L1 and anti-CTLA-4. According to the NWA, chemotherapy improves PFS and OS more than durvalumab with tremelimumab. No appreciable development in the HNSCC patients’ survival rates was observed in any of the three trials. It appears that more patients may experience a response to chemotherapy in KESTREL [22]. Even though the arm receiving the EXTREME regimen showed a worse median PFS. Nonetheless, these kinds of responses are typically not long-lasting, as evidenced by the fact that patients with PD-L1-high expression who received durvalumab or durvalumab plus tremelimumab had a higher 12-month PFS rate than those who received the EXTREME regimen; additionally, the median DoR and the proportion of patients who remained in response at 12 months were greater in the former group as well as in the latter group when compared to the EXTREME regimen, for both PD-L1-high expression patients and all randomized patients. In addition, survival was numerically lower with the EXTREME regimen in patients who did not receive further immunotherapy compared to durvalumab or durvalumab plus tremelimumab in both the population of patients with PD-L1-high expression and in all randomized patients. These findings suggest that ICIs have a long-term impact on patients.

Similar to this, the CONDOR research [28] focuses on a group of patients with R/M HNSCC who are PD-L1-low/negative and whose illness progresses during or following one platinum-containing regimen. The overall response rate (ORR) was similar across treatment groups and subgroups of patients with low or no PD-L1 expression, showing that durvalumab may be beneficial independent of PD-L1 status. Additionally, evidence of durvalumab-beneficial activity within the EAGLE research [27] included full responders and longer durations of response compared to chemotherapy, indicating that durvalumab’s benefit outlasted chemotherapy’s. However, the monotherapy and combination therapy groups were not compared in the EAGLE research. These phenomena could be caused by the patients’ PD-L1 status, the chemotherapy arm’s medication choice, the subsequent therapy, and differences in the clinical characteristics of the randomized groups. To determine how best to use ICI combination therapy in patients, more investigation is required.

Apart from the above, nivolumab plus ipilimumab were also applied to the clinical trials of advanced HNSCC patients [23]. However, the outcomes did not indicate significant improvement in OS. Nevertheless, the combination of nivolumab and ipilimumab demonstrated a favourable safety profile. Further research is required to investigate pharmaceutical options.

### 4.4 Limitations

A few restrictions on this analysis are as follows: (1) Because of search technique constraints, it was not possible to retrieve matches for all studies. (2) A certain level of racial and age-based clinical variability exists among the groups included in each study, which could have an impact on the stability of the findings. (3) The probability of outcomes is increased for specific therapies, resulting from the limited number of included experiments. (4) The limited evidence for selection bias, performance bias and detection bias also may influence the results of the network meta-analysis.

## 5 Conclusions

The combination of nivolumab and chemotherapy may represent the most effective and affordable course of treatment for advanced HNSCC among all the treatments enrolled in this study. Additionally, for individuals with PD-L1-positive HNSCC, nivolumab with radiation therapy is also a sensible decision. Further investigation is necessary to ascertain the effectiveness of the combination of chemotherapy and pembrolizumab. For individuals with advanced HNSCC, nivolumab and pembrolizumab may represent the most effective monotherapy approach among all the monotherapy therapies enrolled in this study. Durvalumab may be advantageous for those who do not take PD-L1 into account. However, ICI combo treatment does not significantly improve PFS and OS in all HNSCC patients who enrolled in this study.

## Supplementary Materials





## Data Availability

The data of this study can be obtained from the corresponding author according to reasonable requirements.

## Supplementary Materials

The supplementary material is available online at https://www.techscience.com/doi/10.32604/or.2025.065911/s1.
