# Supplementary material for: Efficacy of Immune Checkpoint Inhibitors in the Treatment for Head and Neck Cancer Patients: A Systematic Review and Network Meta-Analysis
Source: Oncol Res. 2025 Aug 28;33(9):2263–78. doi: 10.32604/or.2025.065911 (PMC12408847; doi:10.32604/or.2025.065911)
Supplement: Supplementary file 2 [file OncolRes-33-65911-s002.docx]

**Supplementary Table S1:** Search strategy in PubMed.

| 1 | "Mouth Neoplasms"[Mesh] |
| --- | --- |
| 2 | ((Oral Neoplasm[Title/Abstract]) OR (Oral Cancer[Title/Abstract])) OR (Mouth Cancer[Title/Abstract]) |
| 3 | #1 OR #2 |
| 4 | "Otorhinolaryngologic Neoplasms"[Mesh] |
| 5 | (((Otorhinolaryngologic Neoplasm[Title/Abstract]) OR (Otorhinolaryngeal Cancer[Title/Abstract])) OR (Neoplasm, Otorhinolaryngologic[Title/Abstract])) OR (Otorhinolaryngologic Neoplasm[Title/Abstract]) |
| 6 | #4 OR #5 |
| 7 | "Squamous Cell Carcinoma of Head and Neck"[Mesh] |
| 8 | ((((((((((((((((Head And Neck Squamous Cell Carcinomas[Title/Abstract])) OR (Squamous Cell Carcinoma, Head And Neck[Title/Abstract])) OR (Squamous Cell Carcinoma of the Head and Neck[Title/Abstract])) OR (Head and Neck Squamous Cell Carcinoma[Title/Abstract])) OR (HNSCC[Title/Abstract])) OR (Carcinoma, Squamous Cell of Head and Neck[Title/Abstract])) OR (Squamous Cell Carcinoma of the Larynx[Title/Abstract])) OR (Laryngeal Squamous Cell Carcinoma[Title/Abstract])) OR (Squamous Cell Carcinoma of Larynx[Title/Abstract])) OR (Squamous Cell Carcinoma of the Nasal Cavity[Title/Abstract])) OR (Oral Tongue Squamous Cell Carcinoma[Title/Abstract])) OR (Hypopharyngeal Squamous Cell Carcinoma[Title/Abstract]) OR (Oral Squamous Cell Carcinoma[Title/Abstract])) OR (Oral Cavity Squamous Cell Carcinoma[Title/Abstract])) OR (Oral Squamous Cell Carcinomas[Title/Abstract])) OR (Squamous Cell Carcinoma of the Mouth[Title/Abstract])) OR (Oropharyngeal Squamous Cell Carcinoma[Title/Abstract]) |
| 9 | #7 OR #8 |
| 10 | #3 OR #6 OR #9 |
| 11 | "Immunotherapy"[Mesh] |
| 12 | Immunotherapies[Title/Abstract] |
| 13 | #11 OR #12 |
| 14 | "Immune Checkpoint Inhibitors"[Mesh] |
| 15 | (((((((((((((((((((((((((((((((((((((((((((((((((((((((((((((((((((((((((((((((((((((Checkpoint Inhibitors, Immune[Title/Abstract])) OR (Immune Checkpoint Inhibitor[Title/Abstract])) OR (Checkpoint Inhibitor, Immune[Title/Abstract])) OR (Immune Checkpoint Blockers[Title/Abstract])) OR (Checkpoint Blockers, Immune[Title/Abstract])) OR (Immune Checkpoint Blockade[Title/Abstract])) OR (Checkpoint Blockade, Immune[Title/Abstract])) OR (Immune Checkpoint Inhibition[Title/Abstract])) OR (Checkpoint Inhibition, Immune[Title/Abstract])) OR (PD-L1 Inhibitors[Title/Abstract])) OR (PD L1 Inhibitors[Title/Abstract])) OR (PD-L1 Inhibitor[Title/Abstract])) OR (PD L1 Inhibitor[Title/Abstract])) OR (Programmed Death-Ligand 1 Inhibitors[Title/Abstract])) OR (Programmed Death Ligand 1 Inhibitors[Title/Abstract])) OR (PD-1-PD-L1 Blockade[Title/Abstract])) OR (Blockade, PD-1-PD-L1[Title/Abstract])) OR (PD 1 PD L1 Blockade[Title/Abstract])) OR (CTLA-4 Inhibitors[Title/Abstract])) OR (CTLA 4 Inhibitors[Title/Abstract])) OR (CTLA-4 Inhibitor[Title/Abstract])) OR (CTLA 4 Inhibitor[Title/Abstract])) OR (Cytotoxic T-Lymphocyte-Associated Protein 4 Inhibitors[Title/Abstract])) OR (Cytotoxic T Lymphocyte Associated Protein 4 Inhibitors[Title/Abstract])) OR (Cytotoxic T-Lymphocyte-Associated Protein 4 Inhibitor[Title/Abstract])) OR (Cytotoxic T Lymphocyte Associated Protein 4 Inhibitor[Title/Abstract])) OR (PD-1 Inhibitors[Title/Abstract])) OR (PD 1 Inhibitors[Title/Abstract])) OR (PD-1 Inhibitor[Title/Abstract])) OR (Inhibitor, PD-1[Title/Abstract])) OR (PD 1 Inhibitor[Title/Abstract])) OR (Programmed Cell Death Protein 1 Inhibitor[Title/Abstract])) OR (Programmed Cell Death Protein 1 Inhibitors[Title/Abstract])) OR (anti-PD1[Title/Abstract])) OR (anti-PD-1[Title/Abstract])) OR (CD279[Title/Abstract])) OR (anti-PD-L1[Title/Abstract])) OR (anti-PDL1[Title/Abstract])) OR (CD152[Title/Abstract])) OR (anti-CTLA-4[Title/Abstract])) OR (anti-CTLA4[Title/Abstract])) OR (anti-IDO-1[Title/Abstract])) OR (IDO1 inhibitor[Title/Abstract])) OR (IDO-1 inhibitor[Title/Abstract])) OR (anti-LAG3[Title/Abstract])) OR (anti-LAG-3[Title/Abstract])) OR (LAG3 inhibitor[Title/Abstract])) OR (LAG-3 inhibitor[Title/Abstract])) OR (anti-VISTA[Title/Abstract])) OR (VISTA inhibitor[Title/Abstract])) OR (anti-TIM-3[Title/Abstract])) OR (TIM-3 inhibitor[Title/Abstract])) OR (anti-ICOS[Title/Abstract])) OR (ICOS inhibitor[Title/Abstract])) OR (anti-OX40[Title/Abstract])) OR (OX40 inhibitor[Title/Abstract])) OR (anti-B7-H3[Title/Abstract])) OR (B7-H3 inhibitor'[Title/Abstract])) OR (anti-B7-H4[Title/Abstract])) OR (B7-H4 inhibitor[Title/Abstract])) OR (anti-KIR[Title/Abstract])) OR (KIR inhibitor[Title/Abstract])) OR (atezolizumab[Title/Abstract])) OR (avelumab[Title/Abstract])) OR (camrelizumab[Title/Abstract])) OR (cemiplimab[Title/Abstract])) OR (dostarlimab[Title/Abstract])) OR (durvalumab[Title/Abstract])) OR (ipilimumab[Title/Abstract])) OR (lambrolizumab[Title/Abstract])) OR (nivolumab[Title/Abstract])) OR (pembrolizumab[Title/Abstract])) OR (tremelimumab[Title/Abstract])) OR (ticilimumab[Title/Abstract])) OR (tislelizumab[Title/Abstract])) OR (toripalimab[Title/Abstract])) OR (sintilimab[Title/Abstract])) OR (spartalizumab[Title/Abstract])) OR (epacadostat[Title/Abstract])) OR (linrodostat[Title/Abstract])) OR (navoximod[Title/Abstract])) OR (indoximod[Title/Abstract])) OR (relatlimab[Title/Abstract])) OR (lirilumab[Title/Abstract])) OR (tiragolumab[Title/Abstract]) |
| 16 | #14 OR #15 |
| 17 | "Neoadjuvant Therapy"[Mesh] |
| 18 | (((((((((((((new adjuvant therapy[Title/Abstract]) OR (neoadjuvant therapy[Title/Abstract])) OR (Neoadjuvant Chemoradiotherapy[Title/Abstract])) OR (Neoadjuvant Chemoradiation Treatment[Title/Abstract])) OR (Neoadjuvant Chemoradiation[Title/Abstract])) OR (Neoadjuvant Radiotherapy[Title/Abstract])) OR (Neoadjuvant Radiation Treatment[Title/Abstract])) OR (Neoadjuvant Radiation Therapy[Title/Abstract])) OR (Neoadjuvant Radiation[Title/Abstract])) OR (Neoadjuvant Chemotherapy[Title/Abstract])) OR (Neoadjuvant Chemotherapy Treatment[Title/Abstract])) OR (Neoadjuvant Systemic Therapy[Title/Abstract])) OR (Neoadjuvant Systemic Treatment[Title/Abstract])) |
| 19 | #17 OR #18 |
| 20 | #13 OR #16 OR #19 |
| 21 | #10 AND #20 |

**Supplementary Table S2:** Search strategy in Embase.

| #1 | 'mouth tumor'/exp OR 'mouth tumor' |
| --- | --- |
| #2 | 'mouth neoplasm':ti,ab OR 'oral cancer':ti,ab OR 'mouth cancer':ti,ab |
| #3 | #1 OR #2 |
| #4 | 'ear nose throat tumor'/exp |
| #5 | 'otorhinolaryngeal cancer':ti,ab OR 'otorhinolaryngologic neoplasm':ti,ab |
| #6 | #4 OR #5 |
| #7 | 'head and neck squamous cell carcinoma'/exp |
| #8 | 'head and neck squamous cell carcinomas':ti,ab OR 'squamous cell carcinoma, head and neck':ti,ab OR 'squamous cell carcinoma of the head and neck':ti,ab OR 'head and neck squamous cell carcinoma':ti,ab OR 'hnscc':ti,ab OR 'carcinoma, squamous cell of head and neck':ti,ab OR 'squamous cell carcinoma of the larynx':ti,ab OR 'laryngeal squamous cell carcinoma':ti,ab OR 'squamous cell carcinoma of larynx':ti,ab OR 'squamous cell carcinoma of the nasal cavity':ti,ab OR 'oral tongue squamous cell carcinoma':ti,ab OR 'hypopharyngeal squamous cell carcinoma':ti,ab OR 'oral squamous cell carcinoma':ti,ab OR 'oral cavity squamous cell carcinoma':ti,ab OR 'oral squamous cell carcinomas':ti,ab OR 'squamous cell carcinoma of the mouth':ti,ab OR 'oropharyngeal squamous cell carcinoma':ti,ab |
| #9 | #7 OR #8 |
| #10 | #3 OR #6 OR #9 |
| #11 | 'immune checkpoint inhibitor'/exp |
| #12 | 'checkpoint inhibitors, immune':ti,ab OR 'immune checkpoint inhibitor':ti,ab OR 'checkpoint inhibitor, immune':ti,ab OR 'immune checkpoint blockers':ti,ab OR 'checkpoint blockers, immune':ti,ab OR 'immune checkpoint blockade':ti,ab OR 'checkpoint blockade, immune':ti,ab OR 'immune checkpoint inhibition':ti,ab OR 'checkpoint inhibition, immune':ti,ab OR 'pd-l1 inhibitors':ti,ab OR 'pd l1 inhibitors':ti,ab OR 'pd-l1 inhibitor':ti,ab OR 'pd l1 inhibitor':ti,ab OR 'programmed death-ligand 1 inhibitors':ti,ab OR 'programmed death ligand 1 inhibitors':ti,ab OR 'pd-1-pd-l1 blockade':ti,ab OR 'blockade, pd-1-pd-l1':ti,ab OR 'pd 1 pd l1 blockade':ti,ab OR 'ctla-4 inhibitors':ti,ab OR 'ctla 4 inhibitors':ti,ab OR 'ctla-4 inhibitor':ti,ab OR 'ctla 4 inhibitor':ti,ab OR 'cytotoxic t-lymphocyte-associated protein 4 inhibitors':ti,ab OR 'cytotoxic t lymphocyte associated protein 4 inhibitors':ti,ab OR 'cytotoxic t-lymphocyte-associated protein 4 inhibitor':ti,ab OR 'cytotoxic t lymphocyte associated protein 4 inhibitor':ti,ab OR 'pd-1 inhibitors':ti,ab OR 'pd 1 inhibitors':ti,ab OR 'pd-1 inhibitor':ti,ab OR 'inhibitor, pd-1':ti,ab OR 'pd 1 inhibitor':ti,ab OR 'programmed cell death protein 1 inhibitor':ti,ab OR 'programmed cell death protein 1 inhibitors':ti,ab OR 'anti-pd1':ti,ab OR 'anti-pd-1':ti,ab OR 'cd279':ti,ab OR 'anti-pd-l1':ti,ab OR 'anti-pdl1':ti,ab OR 'cd152':ti,ab OR 'anti-ctla-4':ti,ab OR 'anti-ctla4':ti,ab OR 'anti-ido-1':ti,ab OR 'ido1 inhibitor':ti,ab OR 'ido-1 inhibitor':ti,ab OR 'anti-lag3':ti,ab OR 'anti-lag-3':ti,ab OR 'lag3 inhibitor':ti,ab OR 'lag-3 inhibitor':ti,ab OR 'anti-vista':ti,ab OR 'vista inhibitor':ti,ab OR 'anti-tim-3':ti,ab OR 'tim-3 inhibitor':ti,ab OR 'anti-icos':ti,ab OR 'icos inhibitor':ti,ab OR 'anti-ox40':ti,ab OR 'ox40 inhibitor':ti,ab OR 'anti-b7-h3':ti,ab OR 'b7-h3 inhibitor':ti,ab OR 'anti-b7-h4':ti,ab OR 'b7-h4 inhibitor':ti,ab OR 'anti-kir':ti,ab OR 'kir inhibitor':ti,ab OR 'atezolizumab':ti,ab OR 'avelumab':ti,ab OR 'camrelizumab':ti,ab OR 'cemiplimab':ti,ab OR 'dostarlimab':ti,ab OR 'durvalumab':ti,ab OR 'ipilimumab':ti,ab OR 'lambrolizumab':ti,ab OR 'nivolumab':ti,ab OR 'pembrolizumab':ti,ab OR 'tremelimumab':ti,ab OR 'ticilimumab':ti,ab OR 'tislelizumab':ti,ab OR 'toripalimab':ti,ab OR 'sintilimab':ti,ab OR 'spartalizumab':ti,ab OR 'epacadostat':ti,ab OR 'linrodostat':ti,ab OR 'navoximod':ti,ab OR 'indoximod':ti,ab OR 'relatlimab':ti,ab OR 'lirilumab':ti,ab OR 'tiragolumab':ti,ab |
| #13 | #11 OR #12 |
| #14 | #10 AND #13 |

**Supplementary Table S3:** Search strategy in Cochrane.

| #1 | MeSH descriptor: [Mouth Neoplasms] explode all trees |
| --- | --- |
| #2 | (Oral Neoplasm):ti,ab,kw OR (Oral Cancer):ti,ab,kw OR (Mouth Cancer):ti,ab,kw |
| #3 | #1 OR #2 |
| #4 | MeSH descriptor: [Mouth Neoplasms] explode all trees |
| #5 | (Otorhinolaryngologic Neoplasm):ti,ab,kw OR (Otorhinolaryngeal Cancer):ti,ab,kw OR (Neoplasm, Otorhinolaryngologic):ti,ab,kw OR (Otorhinolaryngologic Neoplasm):ti,ab,kw |
| #6 | #4 OR #5 |
| #7 | MeSH descriptor: [Squamous Cell Carcinoma of Head and Neck] explode all trees |
| #8 | (Carcinoma, Squamous Cell of Head and Neck):ti,ab,kw OR (Squamous Cell Carcinoma of the Larynx):ti,ab,kw OR (Laryngeal Squamous Cell Carcinoma):ti,ab,kw OR (Squamous Cell Carcinoma of Larynx):ti,ab,kw OR (Squamous Cell Carcinoma of the Nasal Cavity):ti,ab,kw OR (Oral Tongue Squamous Cell Carcinoma):ti,ab,kw OR (Hypopharyngeal Squamous Cell Carcinoma):ti,ab,kw OR (Oral Squamous Cell Carcinoma):ti,ab,kw OR (Oral Cavity Squamous Cell Carcinoma):ti,ab,kw OR (Oral Squamous Cell Carcinomas):ti,ab,kw OR (Squamous Cell Carcinoma of the Mouth):ti,ab,kw OR (Oropharyngeal Squamous Cell Carcinoma):ti,ab,kw |
| #9 | #7 OR #8 |
| #10 | #3 OR #6 OR #9 |
| #11 | MeSH descriptor: [Immune Checkpoint Inhibitors] explode all trees |
| #12 | (Checkpoint Inhibitors, Immune):ti,ab,kw OR (Immune Checkpoint Inhibitor):ti,ab,kw OR (Checkpoint Inhibitor, Immune):ti,ab,kw OR (Immune Checkpoint Blockers):ti,ab,kw OR (Checkpoint Blockers, Immune):ti,ab,kw OR (Immune Checkpoint Blockade):ti,ab,kw OR (Checkpoint Blockade, Immune):ti,ab,kw OR (Immune Checkpoint Inhibition):ti,ab,kw OR (Checkpoint Inhibition, Immune):ti,ab,kw OR (PD-L1 Inhibitors):ti,ab,kw OR (PD L1 Inhibitors):ti,ab,kw OR (PD-L1 Inhibitor):ti,ab,kw OR (PD L1 Inhibitor):ti,ab,kw OR (Programmed Death-Ligand 1 Inhibitors):ti,ab,kw OR (Programmed Death Ligand 1 Inhibitors):ti,ab,kw OR (CTLA-4 Inhibitors):ti,ab,kw OR (CTLA 4 Inhibitors):ti,ab,kw OR (CTLA-4 Inhibitor):ti,ab,kw OR (CTLA 4 Inhibitor):ti,ab,kw OR (Cytotoxic T-Lymphocyte-Associated Protein 4 Inhibitors):ti,ab,kw OR (Cytotoxic T Lymphocyte Associated Protein 4 Inhibitors):ti,ab,kw OR (Cytotoxic T-Lymphocyte-Associated Protein 4 Inhibitor):ti,ab,kw OR (Cytotoxic T Lymphocyte Associated Protein 4 Inhibitor):ti,ab,kw OR (PD-1 Inhibitors):ti,ab,kw OR (PD 1 Inhibitors):ti,ab,kw OR (PD-1 Inhibitor):ti,ab,kw OR (Inhibitor, PD-1):ti,ab,kw OR (PD 1 Inhibitor):ti,ab,kw OR (Programmed Cell Death Protein 1 Inhibitor):ti,ab,kw OR (Programmed Cell Death Protein 1 Inhibitors):ti,ab,kw OR (anti-PD1):ti,ab,kw OR (anti-PD-1):ti,ab,kw OR (CD279):ti,ab,kw OR (anti-PD-L1):ti,ab,kw OR (anti-PDL1):ti,ab,kw OR (CD152):ti,ab,kw OR (anti-CTLA-4):ti,ab,kw OR (anti-CTLA4):ti,ab,kw OR (anti-IDO-1):ti,ab,kw OR (IDO1 inhibitor):ti,ab,kw OR (IDO-1 inhibitor):ti,ab,kw OR (anti-LAG3):ti,ab,kw OR (anti-LAG-3):ti,ab,kw OR (LAG3 inhibitor):ti,ab,kw OR (LAG-3 inhibitor):ti,ab,kw OR (anti-VISTA):ti,ab,kw OR (VISTA inhibitor):ti,ab,kw OR (anti-TIM-3):ti,ab,kw OR (TIM-3 inhibitor):ti,ab,kw OR (anti-ICOS):ti,ab,kw OR (ICOS inhibitor):ti,ab,kw OR (anti-OX40):ti,ab,kw OR (atezolizumab):ti,ab,kw OR (avelumab):ti,ab,kw OR (camrelizumab):ti,ab,kw OR (cemiplimab):ti,ab,kw OR (dostarlimab):ti,ab,kw OR (durvalumab):ti,ab,kw OR (ipilimumab):ti,ab,kw OR (lambrolizumab):ti,ab,kw OR (nivolumab):ti,ab,kw OR (pembrolizumab):ti,ab,kw OR (tremelimumab):ti,ab,kw OR (ticilimumab):ti,ab,kw OR (tislelizumab):ti,ab,kw OR (toripalimab):ti,ab,kw OR (sintilimab):ti,ab,kw OR (spartalizumab):ti,ab,kw OR (epacadostat):ti,ab,kw OR (linrodostat):ti,ab,kw OR (navoximod):ti,ab,kw OR (indoximod):ti,ab,kw OR (relatlimab):ti,ab,kw OR (lirilumab):ti,ab,kw OR (tiragolumab):ti,ab,kw |
| #13 | #11 OR #12 |
| #14 | #10 AND #13 |

**Supplementary Table S4:** Relative effects of different nonpharmacological interventions to improve PFS for HNC patients.

| **1** | 0.71 (0.43, 1.25) | 1.08 (0.75, 1.59) | 1.06 (0.73, 1.53) | 0.87 (0.55, 1.42) | **0.51 (0.28, 0.92)** | 1.41 (0.89, 2.18) | 0.82 (0.59, 1.15) | 0.81 (0.51, 1.23) |
| --- | --- | --- | --- | --- | --- | --- | --- | --- |
| 1.41 (0.80, 2.33) | **10** | 1.52 (0.99, 2.29) | 1.50 (0.93, 2.25) | 1.23 (0.59, 2.44) | 0.72 (0.32, 1.54) | 1.99 (0.95, 3.83) | 1.16 (0.60, 2.12) | 1.14 (0.54, 2.16) |
| 0.93 (0.63, 1.32) | 0.66 (0.44, 1.01) | **2** | 0.98 (0.72, 1.28) | 0.81 (0.44, 1.46) | **0.47 (0.23, 0.93)** | 1.31 (0.71, 2.27) | 0.76 (0.46, 1.24) | 0.75 (0.41, 1.28) |
| 0.94 (0.66, 1.37) | 0.67 (0.44, 1.08) | 1.02 (0.78, 1.38) | **3** | 0.83 (0.46, 1.51) | **0.48 (0.24, 0.96)** | 1.33 (0.74, 2.35) | 0.78 (0.48, 1.28) | 0.76 (0.43, 1.33) |
| 1.14 (0.71, 1.83) | 0.81 (0.41, 1.69) | 1.23 (0.68, 2.28) | 1.21 (0.66, 2.19) | **4** | 0.58 (0.27, 1.23) | 1.61 (0.85, 2.98) | 0.94 (0.53, 1.67) | 0.92 (0.47, 1.71) |
| **1.96 (1.09, 3.53)** | 1.39 (0.65, 3.15) | **2.11 (1.07, 4.29)** | **2.07 (1.04, 4.14)** | 1.71 (0.81, 3.66) | **5** | **2.76 (1.32, 5.70)** | 1.61 (0.83, 3.17) | 1.58 (0.76, 3.23) |
| 0.71 (0.46, 1.12) | 0.50 (0.26, 1.05) | 0.76 (0.44, 1.41) | 0.75 (0.43, 1.36) | 0.62 (0.34, 1.18) | **0.36 (0.18, 0.76)** | **6** | 0.58 (0.34, 1.03) | 0.57 (0.31, 1.06) |
| 1.22 (0.87, 1.69) | 0.86 (0.47, 1.66) | 1.31 (0.81, 2.19) | 1.29 (0.78, 2.10) | 1.06 (0.60, 1.90) | 0.62 (0.32, 1.21) | 1.71 (0.97, 2.95) | **8** | 0.99 (0.63, 1.46) |
| 1.24 (0.81, 1.95) | 0.87 (0.46, 1.84) | 1.33 (0.78, 2.45) | 1.31 (0.75, 2.35) | 1.08 (0.58, 2.11) | 0.63 (0.31, 1.32) | 1.74 (0.95, 3.27) | 1.01 (0.68, 1.59) | **9** |

Note: 1. chemotherapy; 2. durvalumab; 3. durvalumab + tremelimumab; 4. nivolumab; 5. nivolumab + chemotherapy; 6. nivolumab + ipilimumab; 7. nivolumab + radiotherapy; 8. pembrolizumab; 9. pembrolizumab + chemotherapy; 10. tremelimumab.

**Supplementary Table S5:** Relative effects of different nonpharmacological interventions to improve OS for HNC patients.

| **1** | 0.7 (0.50, 1.01) | 0.95 (0.76, 1.20) | 1.03 (0.83, 1.28) | **0.67 (0.48, 0.93)** | **0.49 (0.29, 0.81)** | 0.96 (0.72, 1.30) | 0.6 (0.24, 1.50) | 0.81 (0.65, 1.00) | **0.71 (0.54, 0.93)** |
| --- | --- | --- | --- | --- | --- | --- | --- | --- | --- |
| 1.43 (0.99, 2.01) | **10** | **1.36 (1.01, 1.80)** | **1.47 (1.06, 1.98)** | 0.96 (0.58, 1.54) | 0.70 (0.37, 1.27) | 1.37 (0.86, 2.16) | 0.85 (0.31, 2.29) | 1.15 (0.75, 1.72) | 1.01 (0.64, 1.55) |
| 1.05 (0.83, 1.31) | **0.74 (0.56, 0.99)** | **2** | 1.08 (0.90, 1.29) | 0.71 (0.47, 1.05) | **0.51 (0.29, 0.89)** | 1.01 (0.69, 1.47) | 0.63 (0.24, 1.63) | 0.85 (0.61, 1.15) | 0.74 (0.52, 1.05) |
| 0.97 (0.78, 1.21) | **0.68 (0.51, 0.94)** | 0.92 (0.78, 1.11) | **3** | **0.65 (0.44, 0.96)** | **0.47 (0.27, 0.82)** | 0.93 (0.65, 1.36) | 0.58 (0.22, 1.50) | 0.78 (0.57, 1.06) | **0.69 (0.48, 0.97)** |
| **1.49 (1.07, 2.09)** | 1.04 (0.65, 1.73) | 1.41 (0.96, 2.14) | **1.53 (1.04, 2.29)** | **4** | 0.72 (0.40, 1.33) | 1.42 (0.93, 2.24) | 0.89 (0.38, 2.10) | 1.2 (0.81, 1.79) | 1.05 (0.69, 1.63) |
| **2.05 (1.24, 3.42)** | 1.44 (0.79, 2.70) | **1.95 (1.13, 3.42)** | **2.11 (1.22, 3.67)** | 1.38 (0.75, 2.52) | **5** | **1.97 (1.10, 3.54)** | 1.21 (0.43, 3.56) | 1.65 (0.96, 2.87) | 1.45 (0.82, 2.58) |
| 1.04 (0.77, 1.39) | 0.73 (0.46, 1.17) | 0.99 (0.68, 1.44) | 1.07 (0.74, 1.54) | 0.70 (0.45, 1.07) | **0.51 (0.28, 0.91)** | **6** | 0.62 (0.24, 1.63) | 0.84 (0.58, 1.20) | 0.74 (0.49, 1.10) |
| 1.67 (0.67, 4.23) | 1.18 (0.44, 3.20) | 1.60 (0.61, 4.17) | 1.72 (0.66, 4.49) | 1.12 (0.48, 2.66) | 0.82 (0.28, 2.32) | 1.61 (0.61, 4.22) | **7** | 1.35 (0.52, 3.50) | 1.18 (0.45, 3.11) |
| 1.24 (1.00, 1.55) | 0.87 (0.58, 1.33) | 1.18 (0.87, 1.63) | 1.28 (0.94, 1.75) | 0.84 (0.56, 1.24) | 0.61 (0.35, 1.04) | 1.19 (0.83, 1.74) | 0.74 (0.29, 1.92) | **8** | 0.88 (0.68, 1.13) |
| **1.42 (1.08, 1.86)** | 0.99 (0.64, 1.56) | 1.35 (0.95, 1.93) | **1.46 (1.03, 2.07)** | 0.95 (0.61, 1.46) | 0.69 (0.39, 1.21) | 1.36 (0.91, 2.05) | 0.84 (0.32, 2.21) | 1.14 (0.88, 1.46) | **9** |

Note: 1. chemotherapy; 2. durvalumab; 3. durvalumab + tremelimumab; 4. nivolumab; 5. nivolumab + chemotherapy; 6. nivolumab + ipilimumab; 7. nivolumab + radiotherapy; 8. pembrolizumab; 9. pembrolizumab + chemotherapy; 10. Tremelimumab.
